# Pediatric pituitary adenomas in Northeast Mexico. A follow-up study

**DOI:** 10.1007/s12020-018-1687-0

**Published:** 2018-08-31

**Authors:** Lucia Torres-García, Ricardo M. Cerda-Flores, Marcela Márquez

**Affiliations:** 1 Instituto Mexicano del Seguro Social. UMAE 25, Monterrey, Nuevo Leon Mexico; 2 Universidad Autónoma de Nuevo León, Facultad de Enfermería, Monterrey, Nuevo Leon Mexico; 3 Karolinska Institute, Department Oncology-Pathology, Stockholm, Sweden; 4 Universidad Autónoma de Nuevo León, Facultad de Medicina, Monterrey, Nuevo León Mexico

**Keywords:** Children, Pituitary adenomas, Clinical analysis, Outcome

## Abstract

**Purpose:**

To review incidence, treatment and outcome of pediatric pituitary adenomas (PAs).

**Methods:**

A follow-up study patients with the age of ≤19 years old who were treated from 1995 to 2015 in Mexico.

**Results:**

Out of 1244 diagnosed PA, 43 patients were children (35 females, 8 males) with a mean age of 17.2 years. The majority were macroadenomas (70%) with prolactinomas (PRL) dominating (63%) followed by non-functioning adenomas (21%). In total, 40% were diagnosed as invasive. Growth hormone (GH) secreting adenomas, adrenocorticotropic hormone secreting and mixed GH-PRL secreting were rare. The treatment modalities were dopamine agonists and surgery. The average treatment time was 44 months with an average follow-up period of 104 months. Sixty-eight percent (27/40) of the patients had complete response after long time follow-up. Thirty-one percent did not respond to treatment whereof three patients died due to advanced disease and late intervention. The principal causes for treatment failure were treatment resistance, late intervention and poor patient compliance.

**Conclusions:**

Sixty eight percent had complete treatment response without any sign of disease, while ~31% did not respond to treatment or did not comply to follow up/treatment. Optimized early diagnose, treatment methods with early intervention, long time follow-up and with better measures for patient compliance should improve outcomes.

## Introduction

Pituitary adenoma (PA), although rare and often benign, is a challenging disorder for diagnosis and subsequent management [1]. The incidence is 0.1 per million children and the prevalence is up to 1 per 1 million children [2–4].

PA constitute <3% of supratentorial tumors in children and 3–5% of all surgically treated tumors occurring in children [2, 3, 5–9].

In 2004, the WHO published a classification system for pituitary tumors relying on immunohistochemistry, presence/absence of secretory products and other ultrastructural features [10]. Separating benign adenomas and pituitary carcinomas, the classification identified atypical adenomas suggesting aggressive potential, indicated by the presence of invasive growth, high mitotic index, a Ki67 labeling index and extensive nuclear staining for p53 [11]. Classification based on histopathological markers does not always correlate with clinical behavior. Benign typical adenomas may sometimes have early recurrence and show resistance to therapy while atypical adenoma are not always invasive, and do not always recur.

Recent identification of causative mutations of genes regulating pituitary tumorigenesis. Genes involved related to cell signaling are *GNAS, PRKAR1A, and SDHx*, and those related to cell growth and proliferation are *MEN1*, CDKNIB, *AIP, PTTG, TGF-∞, FGFR4, and BMPs*. This is the base for molecular screening of young apparently healthy family members. Gene defects can affect both sporadic and familial cases, familial for about 5% of PA [1].

For prolactinomas initial therapy is generally dopamine agonist. For all other PA, initial therapy is generally transsphenoidal surgery with medical therapy being reserved for those that are not cured by surgery [12]. Craniotomy is rarely performed. In general, surgery results in remission in 80–90% of the cases with microadenomas and 40–70% of those with macroadenomas. Complications after surgery, decrease with experienced surgeons and most prevalent is hypopituitarism (7.2%) and diabetes insipidus (7.6%). Radiation therapy is kept for patients who do not achieve remission after surgery [12].

Macroadenomas at presentation are more common in boys than in girls [12] and they have a higher incidence of neurological and ophthalmological disturbances, e.g., cranial nerve compression, headaches, visual loss, growth or pubertal arrest and other pituitary dysfunctions. Pubertal females generally present with symptoms of pubertal arrest, hypogonadism, and amenorrhea [3, 13, 14].

PA may become symptomatic in childhood, but the majority present with symptoms in adolescence [8]. Adrenocorticotropic tumors (ACTH) are more common before puberty while prolactinomas (PRL) are the most common adenomas in puberty [15]. Non-functioning (NF) adenomas, including silent gonadotrophic cell adenomas, are extremely rare [8, 9, 16, 17]. In adults, NF adenomas predominate. Thyroid-stimulating hormone cell adenomas are exceptionally rare in both children and adults [18]. Growth hormone (GH) cell adenomas are reported to have the most equal distribution in the various age groups and account for about 10% of surgically treated pituitary adenomas (PA) in patients <20 years [17]. Concerning gender, a dominance of females has been noted, which may mostly reflect the relative contribution of the two major types of adenomas, PRL and ACTH adenomas [5, 7]. Majority of macroadenomas has been described especially between PRL cell adenomas [2, 9].

Since PA is rare in children, it is most important to collect all available data to improve the diagnostics and determine optimal management of the disease. The present study is a review of the frequency, pathology and therapeutic outcome of pediatric PA diagnosed from 1995 to 2015 at an endocrinology hospital in Monterrey city, Mexico.

## Patients and methods

This is a follow-up study of pediatric PA diagnosed at Mexican Institute of Social Security (IMSS), UMAE 25 specialist hospital in Monterrey, Mexico between 1995 and 2015.

### Evaluation and diagnostic testing

Patients with a confirmed diagnosis and with complete medical records were selected. The patients were divided according to age as follows: prepubescent (0–11 years), pubescent (12–17 years), and postpubescent (18–19 years).

The diagnosis was based on the following criteria: clinical symptoms, positive magnetic resonance imaging (MRI) scan, biochemical confirmation (radioinmunometric assay), oral glucose tolerance test (OGTT), Insulin-like growth factor 1 (IGF-1) serum concentration, and in surgically treated patients, the diagnosis was confirmed by histopathology.

The tumor volume measurements were based on Hardy-Vezina 1979 classification, as microadenoma ≤ 10 mm diameter and macroadenoma > 10 mm diameter. A normal prolactin value range of 2.64–20 ng/ml, while >20 ng/ml was considered abnormal. Prolactin value <0.1ug/ml was classified as a NF adenoma. Serum IGF-1 was graded according to Tanner staging of puberty [19].

Hormonal evaluation or MRI was used to define remission and recurrence after surgery.

Complete therapy response, was defined as: 12 months with normal hormonal values for prolactin, adrenocorticotropic hormone (ACTH), cortisol, GH, insulin-like growth factor 1 (IGF-1), no visible tumor at MRI scan, and regress of symptoms secondary to hormonal and neurological alterations.

Hypopituitarism was determined based on standard biochemical testing of pituitary function. Low free levothyroxine (T4) with normal or low thyroid-stimulating hormone (TSH) was considered to be central hypothyroidism. Low estradiol and low FSH in women with amenorrhea or menstrual irregularity were considered evidence of hypogonadism in women.

Body mass index (BMI) was based in the WHO BMI classification [20].

Family history of PA was recorded in all the patients.

## Treatment

### Prolactinomas

For micro- and macroprolactinomas, initial therapy was dopamine agonists (DA) to normalize PRL levels and decrease the tumor size and improving/controlling symptoms secondary to hormonal and neurological alterations. The patients with hyperprolactinaemia were given oral bromocriptine (BRC), 2.5 mg each 8–12 h or 0.5-1 mg weekly cabergoline (CBG). If there is not response of the DA, doses were increased to CBG 3 mg weekly or BRC 10 mg by day. If there are resistance to DA treatment with hyperprolactinaemia and was shrinkage to less 50% of the original size, then pituitary surgery was considered. Surgery was used for patients with macroadenomas invasive to the optical chiasm and when there was no response after DA treatment within 4 weeks. Surgery was by the transsphenoidal (TSF) or fronto-temporal (FT) approach. The FT was utilized when the macroadenoma was inaccessible from the TSF route and on microadenomas located below the diaphragma sellae or narrow waist at the diaphragma sellae. A repeated FT surgery was necessary to complete tumor resection. Pituitary radiotherapy (RT) was considered to be the third-line therapy when DA and surgical approaches had failed. Linear RT was given to the hypophysis, 2–3 Gy/day during 15 days, whenever the tumors were invasive, or when surgery and dopamine agonist (DA) failed. Chemotherapy was used in one aggressive PRL case (no data available on which chemotherapy drug that was used).

### GH-secreting adenomas

Medical therapy was required for a biochemical control for patients DA for hyperprolactinaemia in combination with high doses of LAR (20 mg/month). Macroadenomas surgery first-line option was by the TSF or FT approach. Invasive macroadenomas required medical therapy and/or radiotherapy after surgery. Linear RT was given to the hypophysis, 2–3 Gy/day during 15 days. Gamma-knife radiosurgery procedure was given in a private USA clinic, with a margin dose 18 Gy for tumor difficult access.

### NF adenomas

Macroadenomas surgery was by the TSF or FT (when the macroadenoma was inaccessible from the TSF route) was the general first approach. A repeated FT surgery was necessary to complete tumor resection. Linear radiotherapy was given to the hypophysis, 2–3 Gy/day during 15 days. DA medical therapy was recommended if is required. Chemotherapy was used in one aggressive NF case (no data available on which chemotherapy drug that was used).

### PRL/GH-secreting adenomas

Macroadenoma TSF surgery was necessary. Dopamine agonist and LAR medical therapy was required in combination with linear RT procedure.

### Corticothoph (ACTH-Secreting) adenomas

We recommended macroadenoma surgery as first-line option and LAR medical therapy. But the patient refused therapy.

### Ethical approval

All procedures performed in the studies involving human participants were in accordance with the ethical standards of the institutional committee and with the “1064 Helsinki Declaration” and its later amendments or comparable ethical standards.

### Data analysis

Statistical analysis was performed using the statistical package IBM SPSS v24. Mean comparisons (paired Student’s *t*-test) between the macro- and microadenomas of PRL values were done. To know the sample size, the software nQuery Advisor v4.0 for paired t-test of mean difference to zero was used. For both adenomas types, the significant level was 0.05, two-sided test and a power of 98% were applied. For macroadenomas (*n* = 26), the PRL means found before was 187.92 and after 24.50 with a mean difference of 163.42, standard deviation of differences of 196.73, and effect size of 0.831. For microadenomas (*n* = 13), the PRL means found before was 188.53 and after 12.74 with a mean difference of 175.79, standard deviation of differences of 130.48, and effect size of 1.347. A *p*-value < 0.05 was considered significant.

## Results

### Clinical presentation

Out of 1244 diagnosed patients, 43 (3.4%) were children, 35 girls (81.3%), and 8 boys (18.6%). Two patients refused treatment and were not included in the investigation. The mean follow-up time was 144 months. Pathology: mean age at diagnosis was 17.2 years (pubescent and postpubescent; range 14–19 years). The majority of the patients presented with adenomas at puberty. None of the patients had a family history of PA. They had mostly macroadenomas (70%) with prolactinomas (PRL) dominating (63%) followed by NF adenomas (21%). In total, 40% were diagnosed as invasive and the vast majority was PRL. GH secreting adenomas (12%), adrenocorticotropic hormone (ACTH) secreting, mixed GH/PRL secreting, and non-secretory adenomas were rare or very rare (<3%). Symptoms: The most common presenting symptoms were amenorrhea, galactorrhea (female patients), headache, hypothyroidism, abnormal BMI, and visual disturbance. The symptoms appeared most commonly at puberty. Pediatric PA tumor type distribution of symptoms and signs classified on the basis of tumor secretion are shown in Table 1.

**Table 1:** Pediatric pituitary adenomas clinical characteristics at diagnoses

	PRL	NF	PRL/GH	GH	ACTH	Total
	*N*=27	*N*=9	*N*=1	*N*=5	*N*=1	*N*=43
Sex (m/f)	2/25	3/6	0/1	2/3	1/0	8/35
Mean age	18	17	19	16	18	17.6
Tumor size (macro/micro)	16/11	7/2	1/0	5/0	1/0	17/26
Invation yes/no	6/21	6/3	0/1	4/1	1/0	17/26
*Inicial symptoms, percent of total number of cases*						
Amenorrhea	18 (44)	0 (0)	0 (0)	1 (2)	0 (0)	19 (46)
Galactorrhea	13 (32)	0 (0)	1 (2)	1 (2)	0 (0)	15 (36)
Headaches	11 (27)	7 (17)	0 (0)	2 (5)	0 (0)	20 (46)
Visual disturbances	10 (23)	3 (7)	0 (0)	3 (7)	0 (0)	16 (39)
Hypothyroidism	6 (14)	2 (5)	0 (0)	2 (5)	0 (0)	10 (23)
Overweight	11 (23)	2 (5)	1 (2)	1 (2)	1 (2)	16 (39)
Obesity	9 (22)	1 (2)	0 (0)	1 (2)	0 (0)	11 (27)
Hypopituitarism	2 (5)	1 (2)	0 (0)	1 (4)	0 (0)	4 (10)
Acromegaly	0 (0)	0 (0)	1 (2)	5 (11)	0 (0)	6 (14)
Diabetes II	1 (2)	0 (0)	0 (0)	0 (0)	0 (0)	1(2)
Diabetes Insipida	0 (0)	1 (2)	0 (0)	0 (0)	0 (0)	1(2)
Nausea	1 (2)	0 (0)	0 (0)	0 (0)	0 (0)	1(2)
Polymenorrhea	1 (2)	0 (0)	0 (0)	0 (0)	0 (0)	1(2)
Arthralgia	0 (0)	0 (0)	0 (0)	1 (2)	0 (0)	1(2)

Symptoms and signs percentages (%) are calculated according from 41 cases retrieved from medical files. In two cases (death), symptoms were not recorded

*N* number of patients, *PRL* prolactinomas, GH growth hormone-secreting adenoma, *NF* non-functioning adenoma, *ACTH* adrenocorticotropic hormone-secreting adenoma

### Treatment

The majority of the patients received medical therapy and some combined with surgery (42%) and radiosurgery (gamma-knife), some also with added radiotherapy (RT and external beam radiation). The surgery techniques were 15/21 cases fronto-temporal and 6/21 cases transsphenoidal surgery. Three patients did not receive treatment: two refuse treatment and a microadenoma NF get spontaneous remission all of them (3/43) were excluded of the outcome list.

### Adverse events

Side effects from the dopamine agonist treatment included nausea, dry mouth, dyspepsia and dizziness. One patient receiving cabergoline had a minor stenosis of the ureter but without affecting renal function. No valvular stenosis was recorded.

### Outcome

The average treatment time for achieving complete response was 41.1 months for prolactinomas, 56.2 months for GH releasing adenomas and 30 months for NF adenomas. Sixty eight percent had complete treatment response without any sign of disease, while ~31% did not respond to treatment or did not comply 5to follow-up/treatment.

All patients had positive MRI scans at base line. Four patients (10%) had positive MRI post-therapy. Six patients (15%) MRI post-therapy data were not available. Of patients with prolactinomas, all had abnormal serum levels at base line. Three patients (7.5%) had abnormal values of prolactin post therapy. The prolactin values after treatment was not available in one patient (2.5%).

Nine cases of the rare type NF adenoma, were found and having bad prognosis. The efficacy of the treatment was shown in decrease of serum PRL values, 93% microadenomas and 87% in macroadenomas. Mean PRL of 13 microadenoma patients was 188.53 ng/ml (basal) and 12.74 ng/ml (after therapy) with significant differences (*t* = 4.858, degree of freedom = 12, *p* = 0.000) between them. Mean PRL values of 26 macroadenoma patients were 187.92 ng/ml (basal) and 24.50 ng/ml (after therapy) with a significant difference (*t* = 4.236, degree of freedom = 25, *p* = 0.000) between them.

### Complications

The cabergoline treatment of the Pat. No. 23 was stopped during pregnancy, causing temporal visual defects during interruption. Two patients had inoperable tumors: Pat. No. 33 tumor was invasive in cavernous sinus with FT surgery fail, due to hemorrhage, RT fail as well, but was eventually successfully treated with gamma-knife radiotherapy. Pat. No. 35 with an invasive tumor involving the carotid artery was deemed surgically inoperable.

One patient with a NF adenoma had transient preoperative diabetes insipidus a rare complication.

Three patients (7.5%) died due to advanced disease: Pat. No. 7, tumor with cerebral stem invasion, Pat. No. 40, late intervention, Pat. No. 20, with a cephalo-rachidien fluid fistula with meningitis complications.

### Statistical analysis

It was not possible to do the correlation analysis for microadenomas and macroadenomas patients between basal or last BMI and PRL values because the size of the sample of BMI was not enough. The BMI for macroadenoma (with a power of 80% and effect size of 0.472) and for microadenoma (with a power of 80% and effect size of 0.488) required, 38 and 35 patients, respectively. Mean BMI values of microadenoma patients were 25.50 (basal) and 23.10 (actual). Mean BMI values of macroadenoma patients were 28.90 (basal) and 27.31 (actual). The prolactin values decreased after treatment 93.2% microadenomas and 89.9% in macroadenomas after 54 months of therapy. The positive treatment impact of PRL was 1379.83% in macroadenomas ((188.53-12.74)/12.74) and 667.02% in microadenomas ((187.92-24.5)/24.50). The patient characteristics, treatments, MRI, BMI, and PRL values and outcome are summarized in Table 2a and b).

**Table 2a:** Prolactinoma patient characteristics, treatment and outcome. Normal prolactin value range of 2.64–20 ng/ml

Pat No.	Age* Yr (Sex)	Tumor	Size	Inva-sion	Surgery Type	Medical+RT	Hormone Replace	BMI Basal	Last BMI	PRL Basal (ng/ml)	Last PRL (ng/ml)	Time Complete response (months)	Time Follow-up (months)	Last MRI (+/-)	Response Yes/Not
1	14 (f)	PRL	I	Y	FT	B	—	18.0	18.0	20.9	1.9	2	144	-	Y
2	16 (f)	PRL	I	N	—	C	T4	27.0	30.6	134.5	13.4	43	144	-	Y
3	16 (f)	PRL	I	N	TSF	B, C	—	17.0	17.0	242.0	0.7	36	84	-	Y
4	16 (f)	PRL	I	N	—	B	T4	28.0	24.0	29.4	17.3	48	156	-	Y
5	16 (f)	PRL	I	N	—	C	—	27.7	20.0	146.0	1.7	48	132	-	Y
8	17 (f)	PRL	I	N	—	C	—	32.8	23.7	370.0	16.0	72	96	-	Y
9	19 (f)	PRL	I	N	—	C	—	24.0	20.0	220.0	10.4	21	72	-	Y
10	19 (f)	PRL	I	N	—	C	T4	30.0	29.0	300.0	7.46	12	84	-	Y
11	19 (f)	PRL	I	N	—	C	PRN	39.0	28.0	310.0	24.0	48	156	-	Y
14	14 (f)	PRL	II	N	—	C, B	—	39.0	39.8	88.7	6.7	14	60	-	Y
15	14 (f)	PRL	II	N	—	C, B	T4	40.8	36.0	112.0	18.0	23	132	-	Y
16	14 (f)	PRL	II	N	—	C	T4	21.0	19.0	60.0	23.4	72	192	-	Y
17	14 (m)	PRL	II	N	FT x 3	C, RT, CT	—	37.7	34.8	38.3	17.6	18	108	-	Y
18	16 (f)	PRL	II	N	—	B	—	27.0	22.0	200.0	12.6	—	120	+	N
19	16 (f)	PRL	III	Y	—	C	T4	29.5	30.5	180.0	8.9	12	144	-	Y
20	17 (f)	PRL	II	N	TSF	C	-	32.0	29.0	500.0	†0.3	—	1	- †	†
21	17 (f)	PRL	II	N	—	C	T4	22.0	20.0	800.0	6.5	84	168	-	Y
22	18 (f)	PRL	II	N	FT	C	-	39.0	24.0	600.0	17.3	—	108	+	N
23	18 (f)	PRL	III	Y	—	C	T4	25.4	24.7	237.3	2.6	36	156	-	Y
24	18 (f)	PRL	II	N	FT	C	T4	30.0	30.0	200.0	0.7	96	192	-	Y
25	18 (f)	PRL	II	N		C	T4	26.0	25.0	350.0	18.7	—	144	ND	N
26	19 (f)	PRL	II	N	FT	C	PDN	31.0	28.0	200.0	12.8	36	168	-	Y
27	19 (f)	PRL	II	N	—	C	T4	23.0	22.0	152.0	12.1	—	84	ND	N
28	19 (f)	PRL	III	Y	—	C	—	26.0	26.0	300.0	128.0	—	84	ND	N
29	19 (m)	PRL	III	Y	FT	C, RT	—	32.0	32.0	400.0	190.0	—	168	ND	N
6	17 (f)	PRL	IV	Y	TSF	C, R, RT	T4	28.0	32.0	371.0	24.8	60	180	-	Y
7	17 (f)	PRL	IV	N	—	C	—	26.0	20.6	56.00	†35.00	—	1	+†	†

**Table 2b:** PRL/GH, GH, NF, and ACTH patient characteristics, treatment and outcome

Pat No.	Age^*^ Yr (sex)	Tumor	Size	Inva-sion	Surgery Type	Medical + RT	Hormone Replace	BMI Basal	Last BMI	PRL Basal (ng/ml)	Last PRL (ng/ml)	Time Complete Response (months)	Time Follow-up (months)	Last MRI (+/-)	Response Yes/Not
30	19 (f)	PRL/GH	II	N	TSF	C, R, RT, LAR	—	26.0	22.0	112.0	10.2	12	60	-	Y
31	15 (f)	GH	III	Y	—	LAR	—	24.5	24.7	3.8	53.9	60	120	ND	ND
32	16 (m)	GH	III	Y	FT	PRT	—	23.8	23.8	PRT	PRT	—	1	PRT	PRT
33	17 (f)	GH	IV	Y	FT, RS	C, R, RT, LAR	T4	24.5	24.7	98.0	0.7	9	84	+	N
34	18 (f)	GH	II	N	FT	—	—	29.0	29.0	29.8	12.8	48	84	-	Y
35	18 (m)	GH	III	Y	TSF	LAR	—	38.5	38.5	6.2	8.3	108	108	-	Y
12	16 (f)	NF	I	N	—	—	T4	14.0	14.0	237.0	2.6	6	36	-	Y
13	17 (f)	NF	I	Y	—	—	—	20.0	23.4	14.0	10.0	6	36	-	Y, SR
36	13 (f)	NF	III	Y	FT	—	GH, PDN, T4	16.7	16.7	59.7	ND	—	60	ND	ND
37	17 (f)	NF	IV	Y	FT x 2	B, RT	—	24.0	25.0	38.8	9.2	—	60	+	N
38	17 (f)	NF	III	Y	FT	—	T4, D, E	29.0	29.0	43.5	19.0	48	144	-	Y
39	18 (m)	NF	III	Y	FT	—	T4	35.0	35.0	84.2	19.0	24	36	-	Y
40	19 (f)	NF	II	N	TSF	C	—	30.0	21.6	ND	†	—	1	†	†
41	19 (m)	NF	II	N	FT	—	T4	23.4	23.4	26.0	19.0	12	48	-	Y
42	19 (m)	NF	III	Y	FT	RT, CT	—	29.0	30.8	24.8	8.0	84	84	-	Y
43	18 (m)	ACTH	III	Y	PRT	PRT	—	32.0	32.0	PRT	PRT	—	96	PRT	PRT

*f* female, *m* male, * at diagnosis, *PRL* prolactinoma, *GH* growth hormone secreting adenoma, *NF* non-functioning adenoma, *ACTH* adrenocorticotropic hormone secreting adenoma, *I* microadenomas, *II* macroadenoma 11–19 mm, *III* macroadenoma 20–29 mm, *IV* macroadenoma ≥ 30 mm, *FT* fronto-temporal, *TSF* transsphenoidal, *RT* radiotherapy, *RS* radiosurgery, *B* bromocriptine, *C* cabergoline, *R* rosiglitazone, *LAR* long acting somatostatin, *CT* chemotherapy, *T4* levothyroxine, *PDN* prednisone, *D* desmopressin, *E* estrogen, *PRT* patient refuse treatment, *ND* no data, *†* death, *SR* spontaneous regression

## Discussion

Accurate information concerning the incidence and prevalence of PA in children and adolescents are deficient owing to its rarity. Although these tumors are very nearly always benign, because of their location (mass effects) and interference with normal pituitary function, morbidity may become significant. Due to this vulnerable and developing patient population, early diagnosis and subsequent sustained therapy are of primary importance.

The present study show some discrepancies compared to previous findings [3, 5]. The cohort had a mean age at diagnosis of 17.2 years (pubescent and postpubescent, range 14–19 years) and the population comprised adolescents but no prepubescent children. In addition, the distribution of pediatric PA, related to secretion, is atypical. The most common types usually encountered at prepubescent age are prolactinomas and ACTH-secreting adenomas. The last category was uncommon in this investigation (1 case). Most common were prolactinomas (63%) and NF (21%). This may be a bias and can be a potential limitation. The reason is that this study was realized in a regional specialist hospital of third level. As pediatric PA occur very infrequently within the pediatric age range, pediatric specialists in Mexico do not have experience in providing accurate diagnosis of PA. For example, ACTH cases may be inaccurately diagnosed as obesity. Mexico have high incidence of obesity problems in children. The pediatric specialist try to solve obesity with diet control while the individual suffer from PA resulting in a late PA diagnose. Mexican pediatric doctors need further education regarding general PA diagnosis, especially in ACTH cases, to improve accurate diagnoses resulting in early therapeutic intervention.

The microadenomas in this study were prolactinomas 9/27 and NF 2/9. Prolactinomas presented typical symptoms, e.g., amenorrhea, polymenorrhea, galactorrhea, hypothyroidism, visual disturbances, headache, and weight gain, similar as reported by Acharya et al. [21]. The symptoms associated with the macroadenomas (e.g., amenorrhea, headache, visual disturbances hypothyroidism) were in accordance as previously reported by Colao et al. [22]. Headache with visual field disturbance was recorded in one patient with suprasellar extension. No case with gynecomastia was recorded; however, in young patients gynecomastia should be carefully investigated since teenagers may not refer to it as a symptom.

Galactorrhea, spontaneous flow of milk from the breasts, occurred in 36% of the patients in the present study, similar as the findings of Cannavo et al. [23].

The most common childhood/adolescence PA is prolactinomas [3, 8, 13, 15] and also in the present study. Sixty three percent were prolactinomas and with female predominance which is also similar in the adult population [3, 4, 18–20, 22, 24].

Regarding micro- or macroadenomas, males are more likely to have macroadenomas at presentation [14, 17, 18, 25, 26] and it was confirmed also in the present study all macroadenomas were in males, 8 out of 43 patients.

One overweight prepubertal patient with an ACTH releasing PA was found. These tumors are rare in the pediatric population and are found most often in prepubescent children [18]. No Nelson or Cushing syndrome patients were found in the current investigation. Cushing tumors are unusual in children [27].

Somatotropinomas GH-secreting adenomas account for 5–15% of pediatric pituitary tumors [9, 13, 17, 24, 28]. Somatotropinomas are commonly macroadenomas at presentation and frequently cause headaches and visual disturbances in addition to the endocrine and metabolic symptoms [13]. Pediatric somatotropinomas is uncommon but usually more aggressive than in adults being invasive in 30–60% of the cases. Acromegaly features are present in 37% of pediatric patients [28]. In the present investigation 12% (5/43) were somatotropinomas (macroadenomas) and 4 of them were invasive. Presenting symptoms were headache, visual disturbance, hypothyroidism, hypopituitarism, and acromegaly.

NF adenomas account for 33–50% of pituitary tumors in some adult’s series, but represent only 4–6% or less of cases in the pediatric patient population [8, 13]. Those tumors are very rare in childhood. Weeb et al. [5] reported 1/20 cases, and Mehrazin et al., 2/21 cases [29]. Haddad et al. [16] and Kane et al. [9] failed to report any non-secretory adenomas. Kunwar et al. [24] and Minerman et al. [18] reported 4/150 and 4/136, respectively. These tumors are frequently macroadenomas at diagnosis presenting growth and/or pubertal failure or with headaches and visual disturbance [13]. In the present study, 7/9 of cases of NF adenomas were macroadenomas. Common symptoms were headache, hypothyroidism, overweight, visual disturbance.

Weight gain and obesity occurring as a result of pituitary tumors or their treatment may lead to significant morbidity and mortality [6, 30]. In the present study, of the prolactinoma patients, 39% were overweight and 27% were obese.

Medical management of prolactinomas with BRC and CBG are typically first line treatment for the normalization of prolactin levels and restoring pituitary function and reducing tumor size [3, 31]. Some authors claim that CBG is more effective and often better tolerated than BRC [4, 22, 25, 32]. Dopamine agonists are effective in reducing tumor size and controlling prolactin levels in 80–90% of microadenomas and about 70% of macroadenomas [31]. In the current investigation, 17 out of 27 treated patients treated with CBG or CBG/BRC were had complete response. It was found that CBG therapy once per week was better tolerated than BRC treatment.

Surgery is especially important in invasive adenomas [29] and for prolactinomas surgery is reserved for patients with acute threat to vision, hydrocephalus, cerebral spinal fluid leak, or for rare tumors that grow despite exposure to increasing doses of dopamine agonists [3, 14, 25]. In the present study, surgery was used for patients with macroadenomas invasive to the optical chiasm and when there was no response after DA treatment within 4 weeks. Fronto-temporal surgery was used in supra or/and parasellar or in optical nerve invasion cases. After surgery, the patients continued low dose CBG as adjuvant treatment to prevent recurrence.

GH secreting tumors are often large and locally invasive and TSF surgery remains the first-line therapy for treating this PA variant [3, 15, 25] and resulting in biochemical normalization in approximately 70–85% of microadenoma patients and in 50% of macroadenomas [15, 25]. This mode of surgery is effective in pediatric patients who show gigantism [15, 28]. Invasive GH adenomas often require multiple treatment modalities additional to surgery to obtain cure [28]. Kunwar et al. [24] suggest that surgery of prolactinomas in pediatric population has good outcome with long-term surgical cure of 82% for all prolactinomas, with very low morbidity and no mortality. Guaraldi et al. [1] did a review of all the suggested as treatments of PA. The standard surgery technique for prolactinomas is TSF, except in small children in whom the sphenoid sinus is not pneumatised [14].

In general PA patients needs to be fully informed of the long time risk of recurrence, and therefore, 68% of the seemingly cured patients, had a prophylactic CBG dose (0.5 mg/week). Long time careful follow up is also recommended. MRI scans and control of hormone levels are essential parts of the long time follow-up procedures.

Concerning the use of radiation therapy for PA, historically radiation therapy used to be adjuvant treatment for recurrent or residual PA. However, its use as adjuvant in these days limited because of its slow rate of hormone normalization and increased incidence of early and late complications [15]. In the case of prolactinomas external radiotherapy is rarely used and only after unsuccessful surgery and/or failing drug therapy [33].

Rarely, pituitary tumors result in metastases, qualifying as pituitary carcinomas according to the latest WHO definition. In the same classification, a subset of tumors with relatively distinct histopathological features were identified and defined as atypical adenomas expected to follow a more aggressive clinical course. In general, aggressive pituitary tumors are typically difficult to manage because of their size, invasiveness, rapid growth, and high frequency of recurrence. These PAs need to be carefully defined clinically and radiologically, identifying histological and molecular markers to identify which patients do have increased risk of early recurrence and disease progression. Currently, no single marker or classification system of pituitary tumor aggressiveness exist. Histology markers as Ki67, p53, 06-methylguanine DNA methyltransferase (MGMT), MSH1, MSH2 can be used to identify “aggressive” pituitary tumors to guide diagnostic and therapeutic decision [11, 34]. Several novel biomarkers have been studied in order to assess their validity as predictive markers of pituitary tumor aggressive behavior. Genomic imbalance has been reported to occur frequently in pituitary tumors [11, 35]. In the present study, 40% of the pituitary tumors were diagnosed as invasive, possible dependent on late diagnoses by the pediatric specialist; however, no histopatological marker was used to study tumor aggressiveness and no genetic studies were done. The PA family history in all these patients was negative.

In summary, the results of this study are that pediatric PA most frequently were macroadenomas (70%) with prolactinomas (PRL) dominating (63%). In total, 40% were diagnosed as invasive (6/27 PRL, 6/9 NF, 4/5 GH and 1/1 ACTH).

The study also shows an unclear decision-making scheme, adapted for late-diagnosis, late-treatment and follow-up. Therefore outcome of medical therapy, surgery, or combined treatments (drug, surgery and radiation therapy or radiosurgery) are not distinguished.

## Conclusion

Although PA is rare in children and adolescents, they need to be carefully diagnosed and identified at an early stage with subsequent therapeutic intervention. Careful documentation of pathology, full diagnostic procedures are needed, including molecular markers in order to identify patients at increased risk of recurrence or subsequent tumor progression. Therapy and follow-up are most important to enhance knowledge of optimal care. Long and sustained treatment with very long follow-up is equally important for a successful outcome. Measures to ensure compliance with sustained therapy and long follow-up needs to be improved in Mexico, which is especially significant with Mexico´s socio-economical diverse population.
